# Supplementary material for: SARS-CoV-2 antibody seroprevalence and associated risk factors in an urban district in Cameroon
Source: Nat Commun. 2021 Oct 6;12:5851. doi: 10.1038/s41467-021-25946-0 (PMC8494753; doi:10.1038/s41467-021-25946-0)
Supplement: Supplementary file 1 — Supplementary Information [file 41467_2021_25946_MOESM1_ESM.pdf]

Supplementary information for:

SARS-CoV-2 antibody seroprevalence and associated risk factors in an urban district in Cameroon

Kenechukwu Nwosu*[1]; Joseph Fokam[2,3]; Franck Wanda[4]; Lucien Mama[5]; Erol Orel[1]; Nicolas Ray[1,6]; Jeanine Meke[4]; Armel Tassegning[4]; Desire Takou[2]; Eric Mimbe[7]; Beat Stoll[1]; Josselin Guillebert MSc[8]; Eric Comte[1,9]; Olivia Keiser[1]; Laura Ciaffi[7,9]

These authors contributed equally: Kenechukwu Nwosu, Joseph Fokam

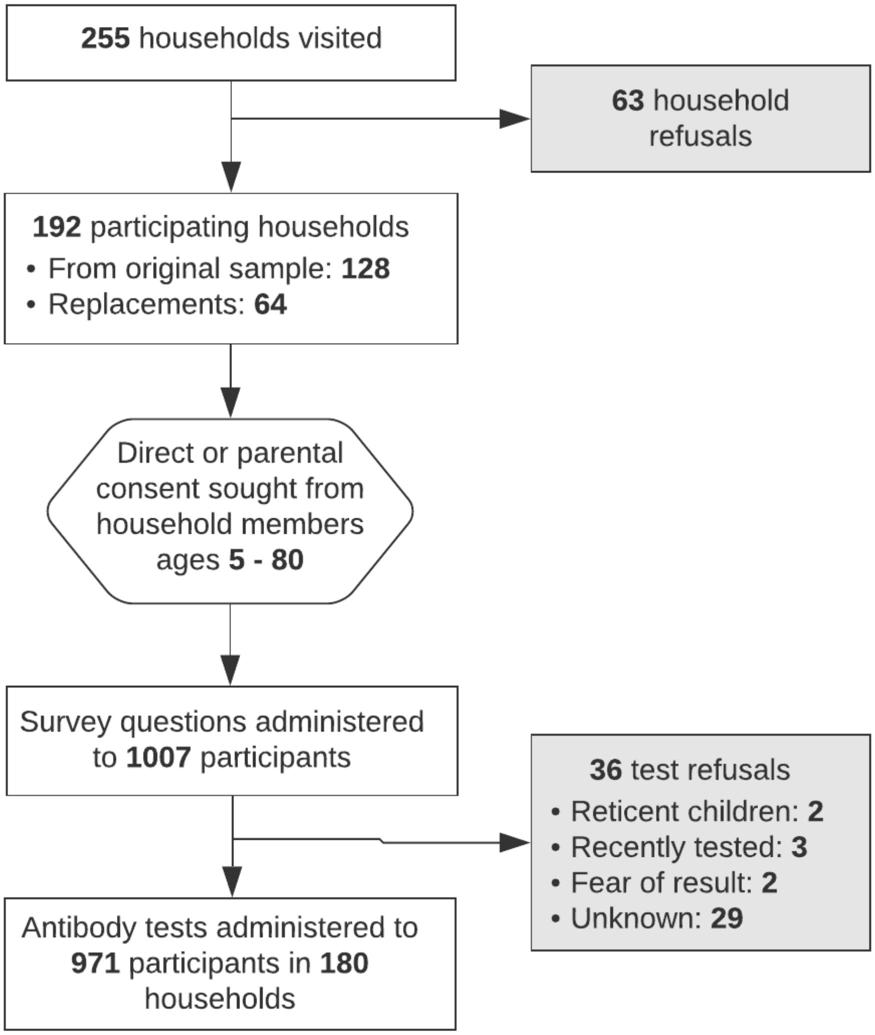

Supplementary Figure 1. Recruitment process and study profile

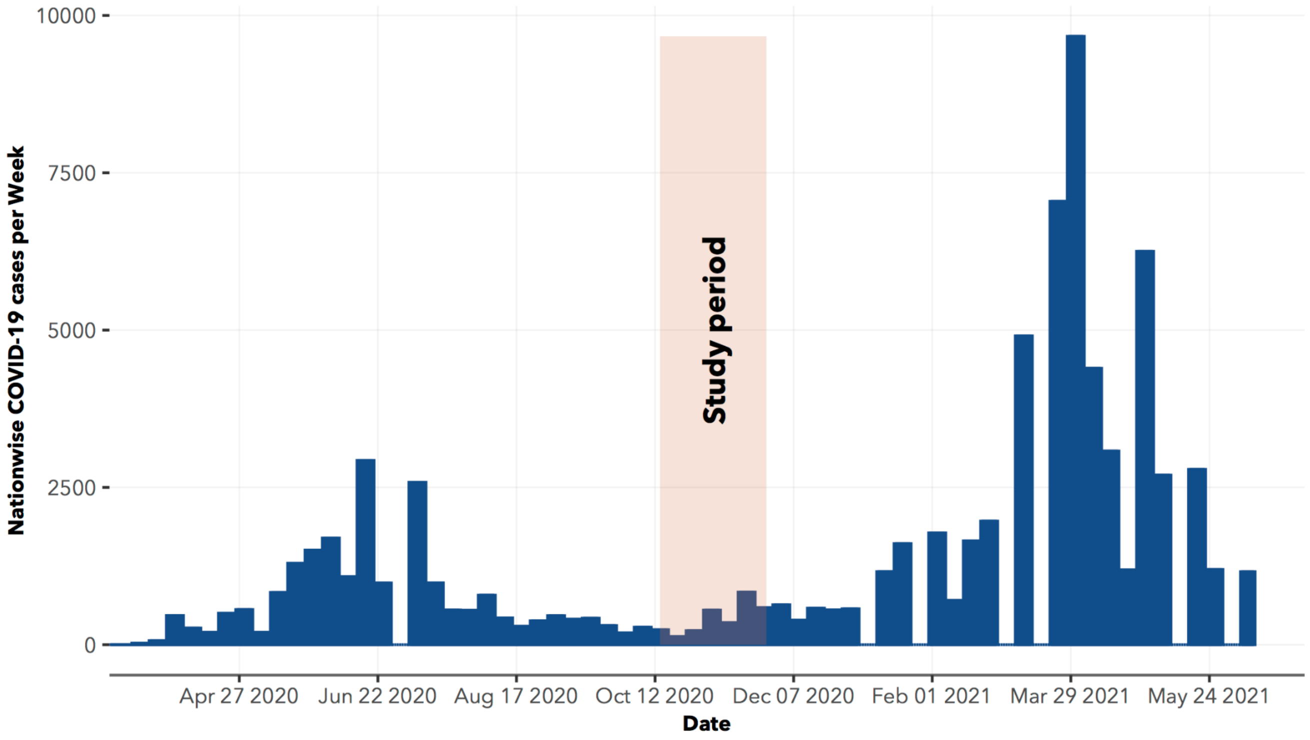

Supplementary Figure 2. Nationally-reported COVID-19 cases counts per week and study sampling period.

Supplementary Table 1. Sample sizes, strata and weights for each age-sex group. DHS stratum sizes refer to the estimated population from the 2018 DHS survey of Yaounde.

Age group	Sex	DHS stratum size	Sample stratum size	Stratum weight	Weight per individual
5 - 14	Female	50155.56	124	404	1.05
5 - 14	Male	48833.29	117	417	1.08
15 - 29	Female	67525.46	187	361	0.937
15 - 29	Male	62723.64	138	455	1.18
30 - 44	Female	44727.47	131	341	0.886
30 - 44	Male	46011.81	81	568	1.47
45 - 64	Female	21278.12	83	256	0.665
45 - 64	Male	21920.72	70	313	0.812
65 +	Female	5645.22	24	235	0.61
65 +	Male	5425.92	16	339	0.88

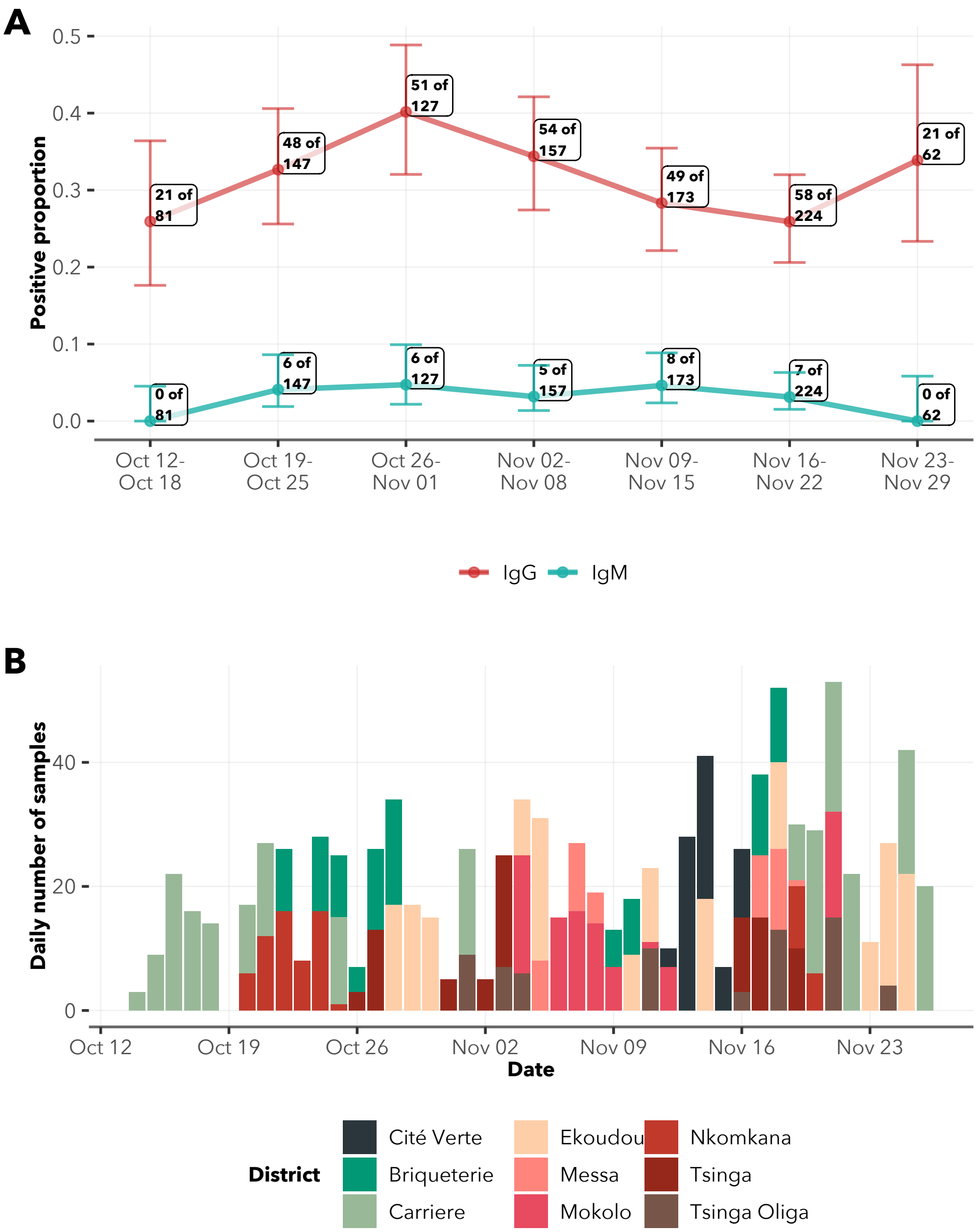

Supplementary Figure 3. Weekly crude seroprevalence and timeline of surveys per neighbourhood. A. Weekly crude IgG and IgM seroprevalence: positive proportion (center) and 95% Wilson confidence interval (error bars). Number positive and sample size is shown for each proportion. **B.** Daily number of samples collected from participants in each district of Cité Verte. (Total count = 971). Note that Cité Verte is the name of one of the neighbourhoods within the Cité Verte health district.)

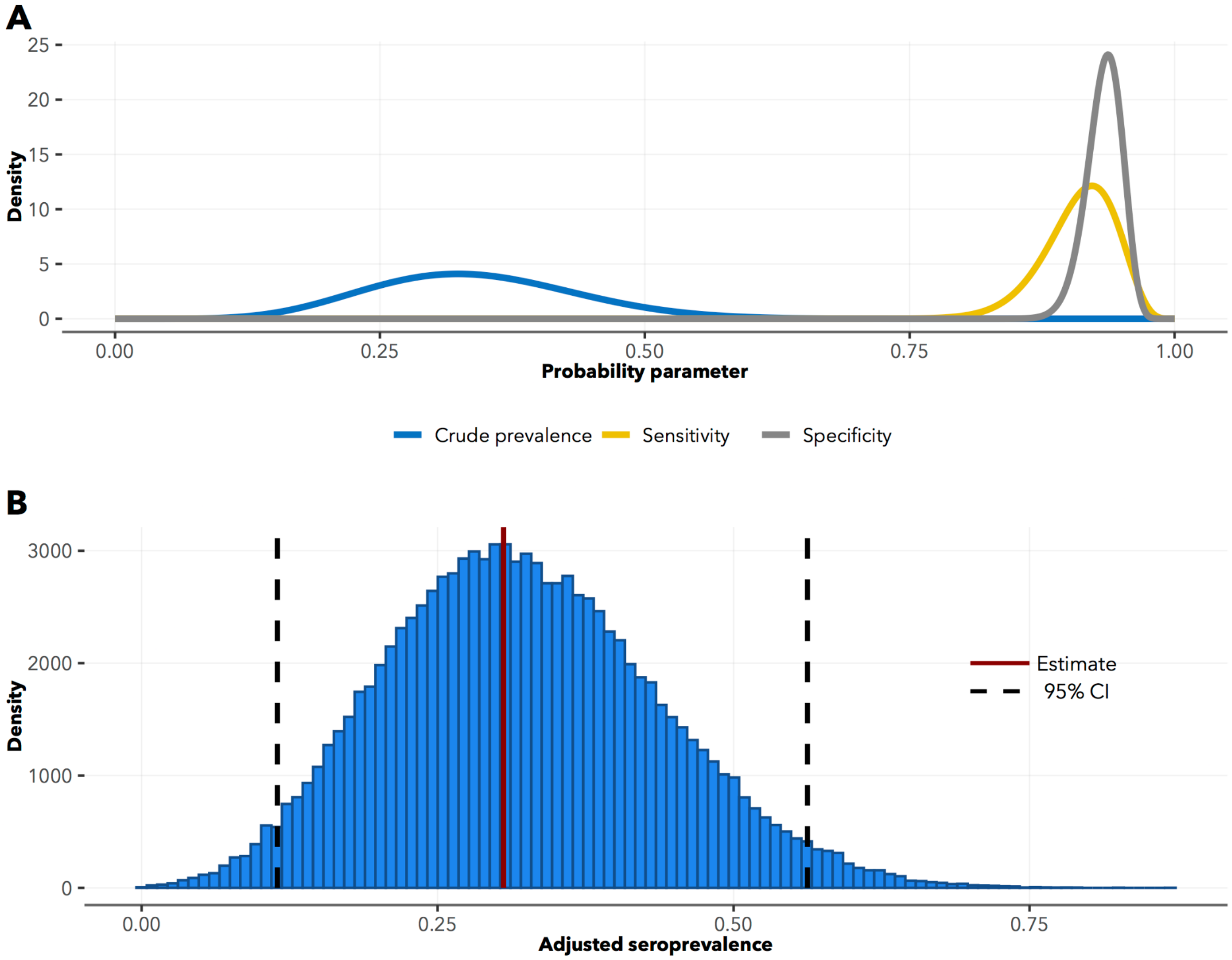

Supplementary Figure 4. Seroprevalence correction. A: Estimated densities of bootstrap parameters—prevalence, sensitivity and specificity—from their 95% confidence intervals. B: Histogram of the sensitivity- & specificity-adjusted seroprevalence estimates from 100,000 bootstrapped samples.

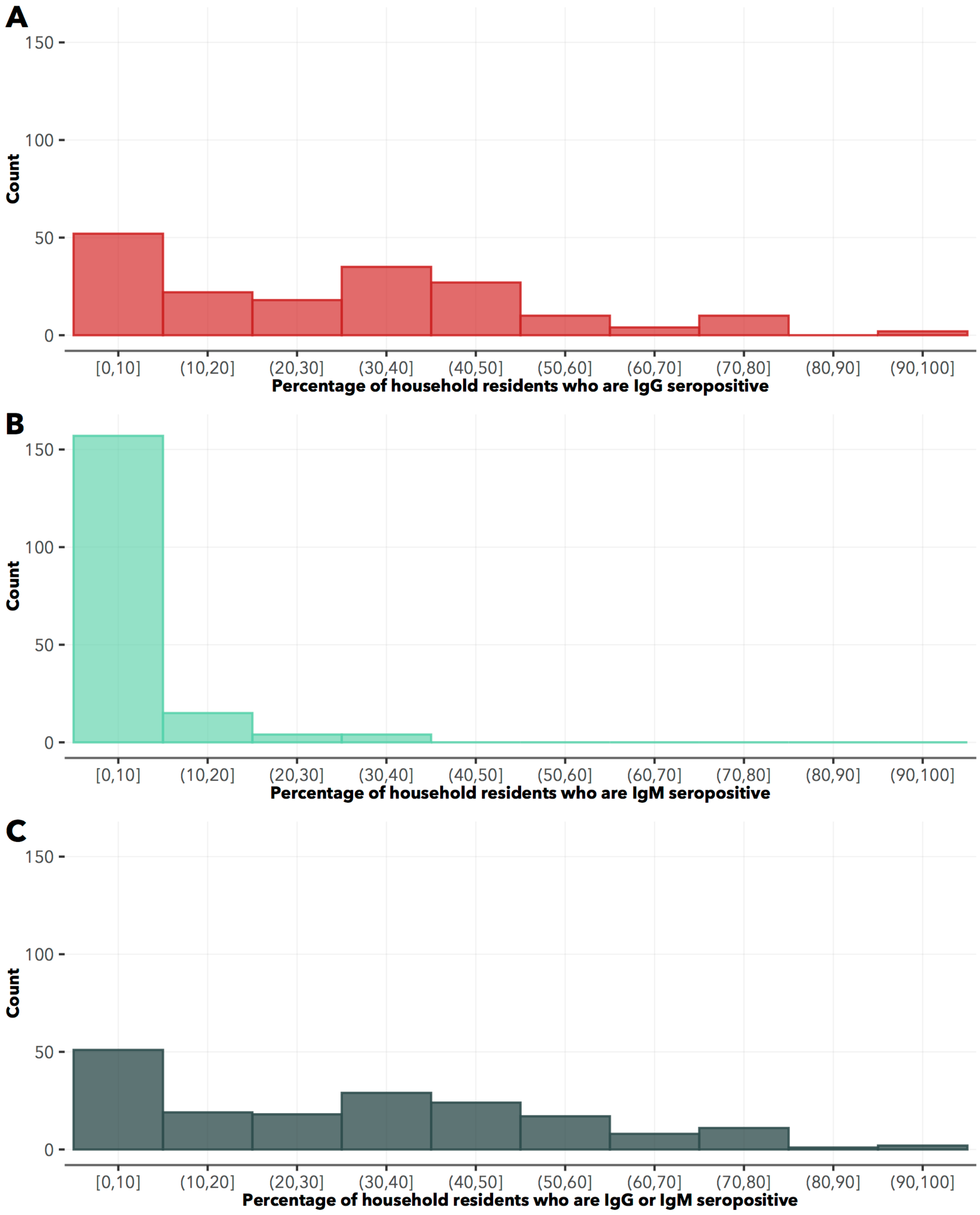

Supplementary Figure 5. Histograms showing the range and distribution of percentage seropositivity across households. A. IgG seropositivity **B.** IgM seropositivity **C.** Combined seropositivity

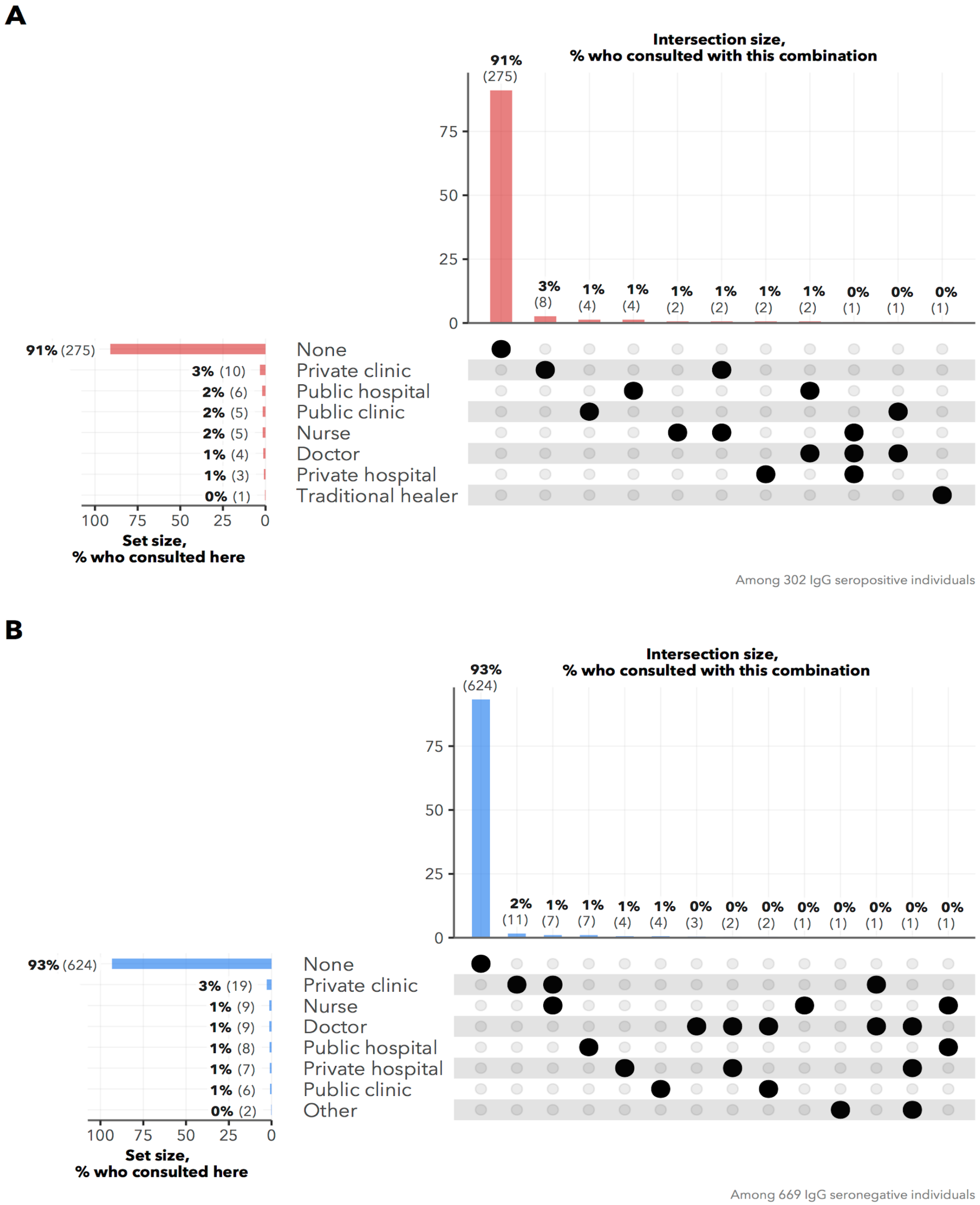

Supplementary Figure 6: Health services used. Health services used by **A.** IgG seropositive and **B.** IgG seronegative individuals, between March 1st 2020 and the date of survey. Horizontal bars to the left represent the percentage of respondents in each group who consulted with the given health service. Vertical bars show the percentage of respondents who consulted with the services symbolized with the black dot(s) in the corresponding column.

Supplementary Methods

Household sampling methodology

The household sampling methodology was based on randomly sampling building objects from a filtered version of the vector-based OpenStreetMap (OSM) data set. We obtained the OSM extract of Cameroun from GeoFabrik (http://download.geofabrik.de/africa.html) on 11 September 2020. After importation of the data in Quantum GIS ver. 3.12 (QGIS), we overlaid it with satellite imagery from BING (Microsoft) online service in order to verify that OSM buildings represented adequately all buildings seen in the imagery, which was the case.

OSM buildings overlapping with the extent of the health district of Cité Verte were first selected. We then filtered all buildings whose OSM building type were deemed not to be residential. The filtered-out buildings were from the following categories: “school”, “university”, “retail”, “publique” (e.g. commissariat, centrale nationale/régional, fédération, etc.), “office”, “no”, “industrial”, “hotel”, “hospital”, “hangar”, “gate”, “farm_auxiliary”, “construction”, “commercial”, “church”. We further filtered out the building named “Palais des congrès” (i.e. congress hall).

The next step consisted in randomly sampling a number of these filtered building objects in each neighborhood, according to the determined household sample size of each neighborhood. Building objects in OSM are sometimes a cluster of adjacent buildings merged into one single building object, especially in dense urban areas. This was the case in several places in the health district of Cité Verte. To facilitate the work of the surveyors, we therefore generated a random point location in each sampled building object, by using the “Random points inside polygons” function of QGIS. The coordinates of these points were exported, numbered, and mapped on top of an urban map of the district to guide the work of the surveyors. A “Standard Operating Procedure” (SOP) document, based on the one developed in Alcoba et al. (2021) and available upon request, was used by the surveyors to guide decisions when a sample household was found not to be residential upon its visit.

Map creation

The map of Cité Verte used was assembled using QGIS version 3.16, based on an extract of a national health area map obtained from the National Mapping Institute of Cameroon. The creation of these health areas is described by Rosencrans et al. (2017)

Reference

1 Alcoba G, Ochoa C, Martins SB, et al. Novel transdisciplinary methodology for cross- sectional analysis of snakebite epidemiology at national scale. PLOS Neglected Tropical Diseases 2021; 15: e0009023.

2 Rosencrans LC, Sume GE, Kouontchou JC, Voorman A, Anokwa Y, Fezeu M, Seaman VY. Mapping for Health in Cameroon: Polio Legacy and Beyond. Journal of Infectious Diseases. 2017 Jul 1;216(suppl_1):S337-S342. doi: 10.1093/infdis/jix008. PMID: 28838181; PMCID: PMC5853277.
