# Supplementary material for: SARS-CoV-2 antibody seroprevalence and associated risk factors in an urban district in Cameroon
Source: Nat Commun. 2021 Oct 6;12:5851. doi: 10.1038/s41467-021-25946-0 (PMC8494753; doi:10.1038/s41467-021-25946-0)
Supplement: Supplementary file 3 — Reporting Summary [file 41467_2021_25946_MOESM3_ESM.pdf]

nature research

Corresponding author(s): Kenechukwu Nwosu

Last updated by author(s): Apr 16, 2021

Reporting Summary

Nature Research wishes to improve the reproducibility of the work that we publish. This form provides structure for consistency and transparency in reporting. For further information on Nature Research policies, see our Editorial Policies and the Editorial Policy Checklist.

Statistics

For all statistical analyses, confirm that the following items are present in the figure legend, table legend, main text, or Methods section.

n/a	Confirmed	
☐	☒	The exact sample size (*n*) for each experimental group/condition, given as a discrete number and unit of measurement
☒	☐	A statement on whether measurements were taken from distinct samples or whether the same sample was measured repeatedly
☐	☒	The statistical test(s) used AND whether they are one- or two-sided<br>*Only common tests should be described solely by name; describe more complex techniques in the Methods section.*
☐	☒	A description of all covariates tested
☐	☒	A description of any assumptions or corrections, such as tests of normality and adjustment for multiple comparisons
☐	☒	A full description of the statistical parameters including central tendency (e.g. means) or other basic estimates (e.g. regression coefficient) AND variation (e.g. standard deviation) or associated estimates of uncertainty (e.g. confidence intervals)
☐	☒	For null hypothesis testing, the test statistic (e.g. *F*, *t*, *r*) with confidence intervals, effect sizes, degrees of freedom and *P* value noted<br>*Give P values as exact values whenever suitable.*
☒	☐	For Bayesian analysis, information on the choice of priors and Markov chain Monte Carlo settings
☒	☐	For hierarchical and complex designs, identification of the appropriate level for tests and full reporting of outcomes
☒	☐	Estimates of effect sizes (e.g. Cohen's *d*, Pearson's *r*), indicating how they were calculated

Our web collection on statistics for biologists contains articles on many of the points above.

Software and code

Policy information about availability of computer code

Data collection	KoBoCollect v1.29.3-1
Data analysis	All code is available at: https://github.com/kendavidn/yaounde_serocovpop_shared<br>Analyses were done with R version 4·0·2.<br>The following R packages were employed in the analysis: cAIC4 (0.9), car (3.0-10), carData (3.0-4), coda (0.19-4), cowplot (1.1.1), DescTools (0.99.40), dplyr (1.0.4), eulerr (6.1.0), forcats (0.5.1), ggallin (0.1.1), ggforestplot (0.1.0), ggnewscale (0.4.5), ggplot2 (3.3.3), ggspatial (1.1.5), ggtext (0.1.1), glmglrt (0.2.2), gridExtra (2.3), gridGraphics (0.5-1), gt (0.2.2), here (1.0.1), highcharter (0.8.2), huxtable (5.2.0), ISOweek (0.6-2), janitor (2.1.0), lattice (0.20-41), leaflet (2.0.4.1), lme4 (1.1-26), lmerTest (3.1-3), lubridate (1.7.9.2), magrittr (2.0.1), Matrix (1.3-2), nlme (3.1-152), packcircles (0.3.4), pacman (0.5.1), paletteer (1.3.0), patch (0.0.1), patchwork (1.1.1), permute (0.9-5), prevalence (0.4.0), purrr (0.3.4), readr (1.4.0), readxl (1.3.1), renv (0.13.0), reshape2 (1.4.4), rjags (4-10), scales (1.1.1), scatterpie (0.1.5), sf (0.9-7), stringi (1.5.3), stringr (1.4.0), tibble (3.0.6), tidyr (1.1.3), tidyverse (1.3.0), usethis (2.0.1), vegan (2.5-7), viridis (0.5.1) and viridisLite (0.3.0)".<br><br>Microsoft BING maps and QGIS 3.16 were used for map creation and processing. A vector-based OpenStreetMap (OSM) data set for Yaounde was obtained from GeoFabrik (http://download.geofabrik.de/africa.html) on 11 September 2020.

For manuscripts utilizing custom algorithms or software that are central to the research but not yet described in published literature, software must be made available to editors and reviewers. We strongly encourage code deposition in a community repository (e.g. GitHub). See the Nature Research guidelines for submitting code & software for further information.

Data

Policy information about availability of data

All manuscripts must include a data availability statement. This statement should provide the following information, where applicable:

- Accession codes, unique identifiers, or web links for publicly available datasets
- A list of figures that have associated raw data
- A description of any restrictions on data availability

Associated raw data for all figures are provided in the source data file. The anonymized participant data can be shared with investigators upon signing of a data access agreement. Requests should be addressed to the corresponding author.

Field-specific reporting

Please select the one below that is the best fit for your research. If you are not sure, read the appropriate sections before making your selection.

☐ Life sciences ☒ Behavioural & social sciences ☐ Ecological, evolutionary & environmental sciences

For a reference copy of the document with all sections, see nature.com/documents/nr-reporting-summary-flat.pdf

Behavioural & social sciences study design

All studies must disclose on these points even when the disclosure is negative.

Study description	Quantitative cross-sectional
Research sample	The population of interest was all inhabitants of Cité Verte, Yaoundé between ages 5 and 80 who were able to sign an informed consent form or had a guardian present who could sign this form. The city of Yaoundé was chosen as it was the putative center of the outbreak in Cameroon, where the majority of cases had been reported.<br><br>255 households were visited, with 192 initially agreeing to participate. In the end, the final research sample consisted of 971 participants from 180 households were tested for antibodies. This sample was determined to be sufficiently large based on the sample size calculations detailed below.<br><br>Women were slightly over-represented; 56.5% of the 971 participants were women. The median age of the sample was 26 years, which is slightly higher than the Cameroonian median of 19 years. This discrepancy is due to the exclusion of children under 5 years old from the study sample.
Sampling strategy	The household sampling methodology was based on randomly sampling building objects from a filtered version of a vector-based OpenStreetMap (OSM) data set obtained from GeoFabrik (http://download.geofabrik.de/africa.html) on 11 September 2020. Based on power calculations with an assumed prevalence of 20%, a precision of 5% and a confidence level of 95%, we estimated a required sample of 245 participants. The final target population was increased to 1000 people (250 households) to further increase statistical power.
Data collection	Data were collected electronically with a KoboCollect form during interviews with study participants. Data collectors were not aware of study hypotheses.<br>Interviews were conducted with one participant at a time. An investigator read out the survey questions to the participant, and the participant's responses were written down. Only the participant(s) and the investigator were present during the interview.
Timing	Data collection took place between October 14, 2020 and November 26, 2020
Data exclusions	One household with four individuals was excluded as they were accidentally interviewed–that is, they were not part of the selected sample.
Non-participation	Of 255 households visited, 194 (75.3%) initially agreed to participate. However, an additional 30 individuals from 12 households did not consent to the antibody test despite having filled the questionnaire. The final sample consisted of 971 participants from 180 households.
Randomization	As this was an observational study, randomization was not applicable.

Reporting for specific materials, systems and methods

We require information from authors about some types of materials, experimental systems and methods used in many studies. Here, indicate whether each material, system or method listed is relevant to your study. If you are not sure if a list item applies to your research, read the appropriate section before selecting a response.

Materials & experimental systems

n/a	Involved in the study
☒	☐ Antibodies
☒	☐ Eukaryotic cell lines
☒	☐ Palaeontology and archaeology
☒	☐ Animals and other organisms
☐	☒ Human research participants
☒	☐ Clinical data
☒	☐ Dual use research of concern

Methods

n/a	Involved in the study
☒	☐ ChIP-seq
☒	☐ Flow cytometry
☒	☐ MRI-based neuroimaging

Human research participants

Policy information about studies involving human research participants

Population characteristics	See above
Recruitment	Based on power calculations with an assumed prevalence of 20%, a precision of 5% and a confidence level of 95%, we estimated a required sample of 245 participants. The final target population was increased to 1000 people (250 households) to further increase statistical power.<br><br>Households were randomly selected from a pre-processed set of residential buildings based on OpenStreetMap data (full procedure in appendix 1 p 7).14 Data collection took place between October 14 and November 26, 2020 (sampling timeline in appendix 1 p 2). In the field, each sampled household was visited by study investigators, who either interviewed residents on the first meeting, or arranged an appointment for a future interview if household members were not all present.<br>In each household, all individuals between five and 80 years of age were included if they (a) had been present in the household for at least 14 days prior to the survey, and (b) could give written informed consent (or had an adult guardian who could give consent).<br><br>Self-selection bias may be present if test refusal correlated with seropositivity. This bias may have artificially inflated or artificially deflated the seroprevalence estimates. Households that think they have had COVID may be less likely to participate in such a study, as they may worry about the stigma of being known to be infected (even though the results are confidential). Alternatively, such households may actually be more eager to participate, since they may want to know whether the COVID-like symptoms they experienced were truly caused by SARS-CoV-2, not another common respiratory virus. There are other possible reasons why self-selection could have biased the results, and we have no strong prior hypotheses about the direction of this effect.
Ethics oversight	The study protocol obtained the ethical clearance (N°2020/09/1292/CE/CNERSH/SP) and the administrative authorization of the Ministry of Health of Cameroon (N°D30-305 845/L/MINSANTE/SG/DROS)

Note that full information on the approval of the study protocol must also be provided in the manuscript.
